# Hantavirus infection in emergency radiology: Imaging manifestations, pathophysiology, and infection control considerations

**DOI:** 10.1007/s10140-026-02499-7

**Published:** 2026-06-22

**Authors:** Platon Braun, Jonathan Lee, Eric D. Cyphers, Ali Gholamrezanezhad

**Affiliations:** 1 Department of Intensive Care Medicine, University Medical Center Hamburg-Eppendorf (UKE), Hamburg, Germany; 2 Department of Radiology, Los Angeles General Medical Center, Los Angeles, CA USA; 3 Department of Radiology, Keck Medicine of USC, Los Angeles, CA USA

**Keywords:** Hantavirus, Andes virus, Hantavirus pulmonary syndrome, Hemorrhagic fever with renal syndrome, Emergency radiology, Infection control

## Abstract

In April 2026, several cases of severe respiratory illnesses were reported aboard the cruise ship MV *Hondius* during its voyage from Argentina. Subsequent laboratory testing identified Andes virus (ANDV), a hantavirus subtype, with rare but documented human-to-human transmission. Although hantavirus infections are uncommon, both hantavirus pulmonary syndrome (HPS) and hemorrhagic fever with renal syndrome (HFRS) are associated with substantial morbidity and mortality. Their rapid progression and potential need for intensive respiratory support continue to pose important challenges for emergency departments, intensive care units, and radiology services. Because pulmonary imaging findings often evolve quickly, radiologists play a critical role in early recognition and triage. This review provides an overview of the pathophysiology, imaging manifestations, and operational considerations relevant to hantavirus infection in emergency radiology practice.

## Introduction

Hantavirus infection is an acute zoonotic disease with significant implications for emergency medicine, critical care, and emergency radiology [1, 2]. These negative-sense, single-stranded RNA viruses within the Hantaviridae family are typically transmitted to humans through the inhalation of aerosolized rodent urine, saliva, or feces [2, 3]. While hantaviruses generally lack the high transmissibility seen in pathogens like COVID-19, the Andes virus (ANDV) variant is unique for its documented potential for human-to-human transmission [1, 4]. The high stakes of this unique transmission were recently brought to light in April 2026 when a cluster of severe respiratory cases emerged aboard the cruise ship MV Hondius. Laboratory testing confirmed ANDV as the causative agent, highlighting the importance of early recognition, exposure history, and infection-control planning in emergency and radiology settings, while recognizing that sustained human-to-human transmission of ANDV remains rare and geographically limited [1, 5].

The clinical significance of hantaviruses is generally categorized into two overlapping syndromic patterns: hemorrhagic fever with renal syndrome (HFRS), which has been described most often in Europe and Asia, and hantavirus pulmonary syndrome (HPS), also termed hantavirus cardiopulmonary syndrome (HCPS), which has been classically associated with viruses in the Americas [2, 6]. This geographic distinction is useful epidemiologically but should not be interpreted as absolute, particularly in the setting of travel-associated exposures and global movement. For consistency with much of the radiology literature, this review uses the term, HPS. Historically, these infections gained prominence during major military conflicts; over 1,000 cases occurred among Finnish and German soldiers in 1942, and approximately 3,200 United Nations soldiers were affected during the Korean War by what was later identified as the Hantaan virus [6]. Later, the 1993 “Four Corners” outbreak led to the identification of the Sin Nombre virus in North America establishing HPS as a fulminant disease with mortality rates reaching 35–40% [2, 6].

Radiologists, particularly those in emergency and thoracic imaging, are often the first to identify the “silent” imaging patterns suggestive of hantavirus infection [7]. The rapid recognition of diffuse non-cardiogenic pulmonary edema, bilateral ground-glass opacities, and pleural effusions, often in the presence of a normal heart size, is vital [1, 2]. In the radiographic series by Ketai et al., interstitial edema was present in 14 of 16 patients, alveolar flooding subsequently developed in 11 of 16, and overall mortality was 43%. Boroja et al. similarly described a fulminant radiographic pattern with rapid progression from mild bilateral interstitial changes to bilateral interstitial and alveolar infiltrates with pleural effusions [8, 9]. These findings directly influence emergency department triage, the initiation of extracorporeal membrane oxygenation (ECMO), and overall patient survival [1, 7]. Consequently, a detailed understanding of the imaging manifestations, differential diagnoses, and stringent infection-control considerations is essential for the modern emergency radiology practice [1, 7].

## Pathophysiology and organ manifestations

### General pathophysiology

The hallmark of hantavirus pathogenesis is a dramatic increase in vascular permeability leading to capillary leak, without direct evidence of cytopathic destruction of the endothelium [3, 10]. Pathogenic hantaviruses are primarily thought to target and infect vascular endothelial cells by utilizing β3 integrins as key entry receptors [3, 11]. This interaction disrupts the endothelial barrier function and impairs vascular integrity [11]. In HFRS, Cosgriff described the central physiologic derangement as vascular dysfunction, manifested by impaired vascular tone and increased vascular permeability, which contributes to shock and renal failure [12]. In HPS, Zaki et al. demonstrated widespread hantaviral antigen in endothelial cells of the microvasculature, particularly in the lung, with fatal cases showing interstitial pneumonitis and, in some cases, diffuse alveolar damage [10]. The resulting disease is largely immunopathological, driven by an intense “cytokine storm” involving high levels of tumor necrosis factor (TNF), interleukin-6 (IL-6), and interferon-gamma (IFN-γ), as well as an overactive T-cell response [3, 13].

### Pulmonary manifestations

The lungs are the primary site of vascular insult in HPS [3, 14]. Increased capillary permeability leads to rapid leakage of protein-rich fluid into the interstitium and alveolar spaces, producing bilateral interstitial opacities that can quickly progress to diffuse alveolar consolidation, often with pleural effusions [3, 13, 14]. The heart typically remains normal in size despite the severity of the pulmonary edema, a key diagnostic feature that could be crucial in emergency radiology [3, 7]. Patients often present with a prodromal phase of fever and myalgia which then shifts abruptly into a cardiopulmonary phase characterized by cough and progressive dyspnea [14, 15]. In the original clinical description of 17 patients, fever and myalgia were present in all patients, cough and gastrointestinal symptoms in 76%, and 15 of 17 patients required mechanical ventilation [14].

### Renal manifestations

Hemorrhagic fever with renal syndrome (HFRS) is characterized by acute kidney injury (AKI) and varying degrees of hemorrhage [3, 12]. Pathogenesis involves viral replication within the renal glomerular and tubular endothelial cells, leading to substantial perinephric and retroperitoneal fluid accumulation and tubular damage [2, 3]. The clinical course typically follows five phases: febrile, hypotensive, oliguric, polyuric, and convalescent [3, 12]. Renal involvement usually begins with sudden proteinuria and hematuria followed by a sharp rise in serum creatinine [2, 3]. In severe HFRS, particularly those caused by the Hantaan or Dobrava viruses, patients may experience intra-abdominal and retroperitoneal hemorrhage. These sequelae can be identified on CT as perinephric fluid collections or generalized renal enlargement [3, 12]. In nephropathia epidemica (NE) which is a milder form of HFRS, abdominal imaging may show renal enlargement, perirenal fascial thickening, and retroperitoneal edema [3, 12, 16].

### Extrapulmonary and systemic manifestations

Beyond pulmonary and renal involvement, hantavirus infection is associated with multisystem manifestations reflecting widespread endothelial dysfunction. Aside from the hallmark pulmonary edema of HPS, many patients suffer from profound myocardial depression and cardiogenic shock, due to the high levels of circulating inflammatory cytokines rather than direct viral infection of myocytes [3, 14]. Moreover, severe abdominal pain, nausea, and vomiting are common, particularly in the prodromal phase. In HFRS, this pain can be so localized and intense and may clinically mimic an acute surgical abdomen, often leading to unnecessary emergency laparotomies before the viral etiology is suspected [3, 12]. Hematologic findings, particularly thrombocytopenia, are nearly universal findings in both HPS and HFRS and serve as a critical early diagnostic marker in the emergency department [3, 14]. Neurologic manifestations such as seizures or encephalopathy, although rare, have been reported in severe hantavirus infections [1, 3]. Key clinical symptoms and laboratory features relevant to emergency radiology triage are summarized in Table 1.

**Table 1:** Clinical and laboratory features relevant to emergency radiology triage in suspected hantavirus infection

Category	Common findings	Relevance for radiologists
Exposure history	Rodent exposure, rural or wilderness exposure, contaminated enclosed spaces, travel to endemic regions, and/or possible ANDV exposure in South America	Raises pre-test probability when imaging shows non-cardiogenic edema or unexplained bilateral opacities
Prodromal symptoms	Fever, myalgias, headache, malaise, abdominal pain, nausea, vomiting, and/or diarrhea	May precede abrupt cardiopulmonary deterioration in HPS
Cardiopulmonary findings	Cough, dyspnea, hypoxemia, hypotension, shock, and/or need for ventilatory support	Supports urgent triage when imaging progresses rapidly
Hematologic findings	Thrombocytopenia, hemoconcentration, and/or leukocytosis	Helps distinguish HPS from routine viral pneumonia or cardiogenic edema
Renal/urinary findings	Proteinuria, hematuria, rising creatinine, oliguria, and/or polyuria during recovery	Supports HFRS or mixed/systemic hantavirus manifestations
Severe disease indicators	Rapidly worsening oxygen requirement, shock, and/or diffuse alveolar disease	Imaging progression may influence escalation of care

### Radiological evaluation of hantavirus infection

#### Imaging modalities

Any imaging modality that minimizes healthcare worker exposure and allows bedside evaluation must be balanced against differences in diagnostic sensitivity and overall clinical information yield. The first-line imaging modality for hantavirus remains chest radiography (CXR), as it is the most accessible and safe imaging modality in emergency settings to track the rapid progression of HPS [8, 9]. CT is not routinely required in all suspected cases but may be useful when radiographs are equivocal, when the diagnosis is uncertain, or when complications and alternative diagnoses are being considered. HRCT is particularly useful for characterizing the nature of pulmonary opacities and identifying early interstitial changes that may be occult on plain films [16, 17].

**Fig. 1:**
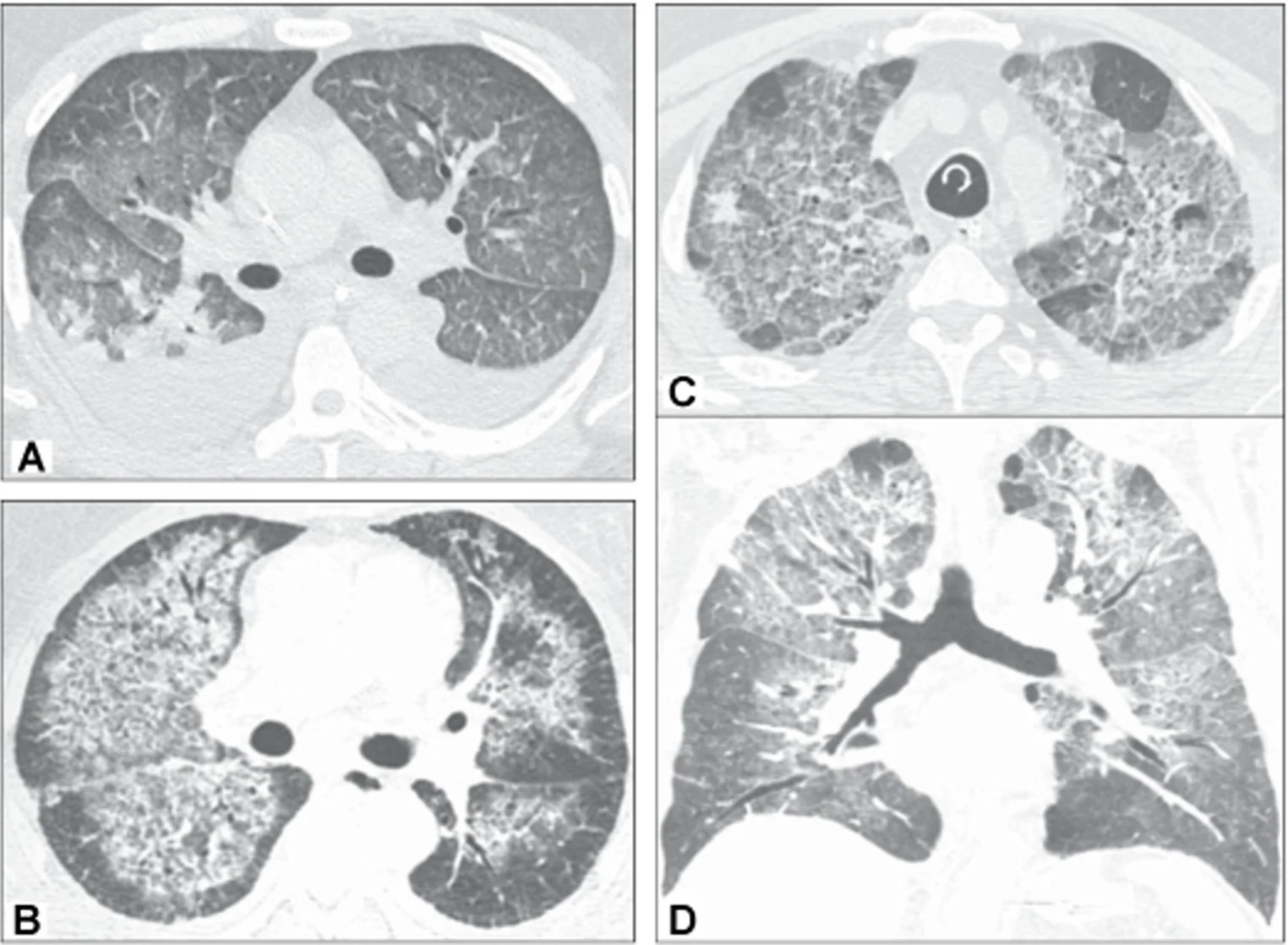
HRCT spectrum of hantavirus pulmonary syndrome (HPS). Axial HRCT image demonstrates bilateral ground-glass opacities and dependent bilateral pleural effusions, consistent with pulmonary capillary leak/non-cardiogenic edema (**a**). Axial CT image demonstrates bilateral ground-glass opacities with smooth septal thickening, reflecting interstitial edema (**b**). Axial and coronal HRCT images demonstrate bilateral ground-glass opacities with superimposed interlobular and intralobular septal thickening, producing a crazy-paving pattern (**c**, **d**). Adapted from Barbosa et al. [17], licensed under Creative Commons Attribution-NonCommercial-ShareAlike 4.0 International. Changes include relabeling and arrangement into a composite figure

The hallmark of HPS is non-cardiogenic pulmonary edema [8]. Key findings include bilateral ground-glass opacities (GGOs), smooth interlobular and intralobular septal thickening, and peribronchovascular thickening [9, 17]. Pleural effusions are frequently observed in severe cases [8, 17]. These findings are illustrated in Fig. 1, which demonstrates the HRCT spectrum of HPS. In HFRS, common findings include renal enlargement and retroperitoneal edema [3]. In HFRS, abdominal and renal findings may predominate, including renal enlargement, perirenal or retroperitoneal edema, and, in severe cases, hemorrhagic complications [12, 16].

#### Role of PET/CT, MRI, and ultrasound

Currently, there is no established routine clinical role for PET/CT or MRI in the acute diagnosis of hantavirus. MRI may be used in rare cases to investigate neurological complications or to characterize renal pathology in HFRS when CT is contraindicated due to acute kidney injury [3, 16]. Acquisition times and the extensive decontamination requirements following imaging may further limit the practicality of MRI in resource-limited settings.

Ultrasound is an effective bedside tool in the emergency department for identifying pleural effusions and monitoring pulmonary edema via B-lines [16]. In HFRS, sonography is the first-line modality for detecting renal swelling, increased cortical echogenicity, and perirenal fluid [3]. The widespread availability of handheld ultrasound devices and the relatively short examination time make ultrasonography an accessible and practical imaging modality in emergency and resource-limited settings.

#### Interpretation of findings

It is paramount to identify progressive imaging findings early to allocate appropriate resources, particularly in high patient-volume settings. Interpretation should focus on temporal disease progression and the absence of classic cardiogenic features.

For suspected HPS, the imaging workflow can be conceptualized as follows:

1. Early Phase: Mild interstitial edema characterized by Kerley B lines and peribronchial cuffing [8].
2. Fulminant Phase: Rapid evolution (often within 24–48 hours) to confluent alveolar consolidation with a central or bibasilar distribution [8, 9].

**Fig. 2:**
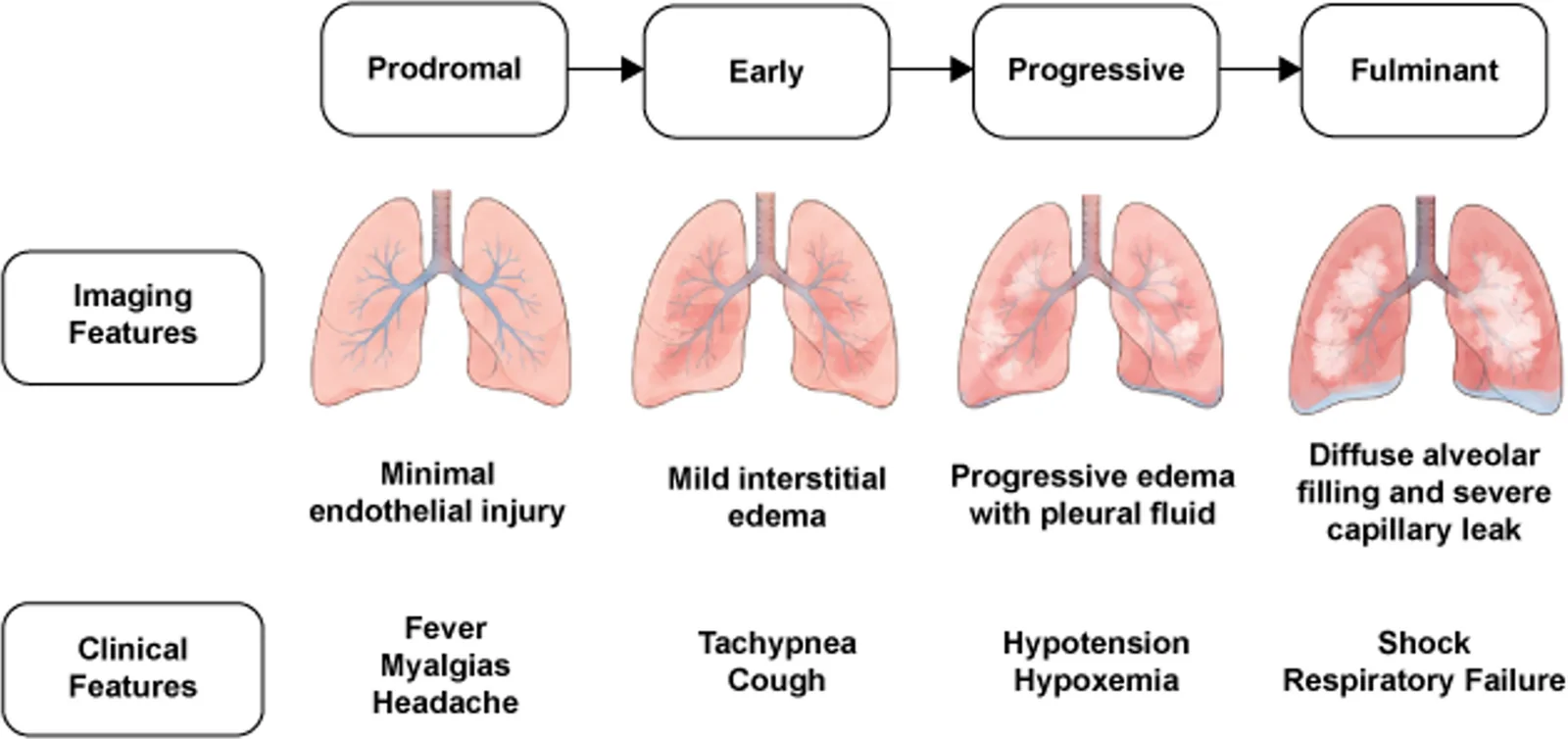
Schematic representation of clinical and imaging progression in hantavirus pulmonary syndrome (HPS). The figure illustrates progression from the prodromal phase to early cardiopulmonary involvement, progressive capillary leak, and fulminant disease, with corresponding imaging and clinical features

A schematic overview of the clinical and imaging progression of HPS is provided in Fig. 2.

The combination of diffuse pulmonary edema, pleural effusions, and a normal heart size may support the diagnosis of HPS when seen in the appropriate clinical and epidemiologic context. This pattern is most useful when accompanied by compatible features such as febrile prodrome, thrombocytopenia, rodent or endemic exposure history, and rapid respiratory decline. However, these imaging findings are not specific and overlap with other causes of non-cardiogenic pulmonary edema.

Because the imaging findings of HPS overlap substantially with other causes of acute bilateral pulmonary opacities, radiologists should avoid diagnosing hantavirus infection on imaging alone. Instead, imaging should be interpreted alongside factors including but not limited to clinical course and exposure history. Table 2 summarizes key imaging mimics and distinguishing clinical clues relevant to emergency radiology practice.

**Table 2:** Imaging differential diagnosis for suspected hantavirus pulmonary syndrome

Entity	Common imaging findings	Helpful distinguishing clinical/laboratory clues
Hantavirus pulmonary syndrome	Rapidly progressive bilateral interstitial edema and ground-glass opacities with often a normal heart size	Febrile prodrome, rodent exposure or endemic travel, thrombocytopenia, hemoconcentration, and/or shock
COVID-19 or other viral pneumonia	Bilateral ground-glass opacities, often peripheral or multifocal and/or consolidation in severe cases	Respiratory viral testing, exposure history, and/or less typical prominent pleural effusions early in the disease process
ARDS	Diffuse bilateral airspace opacities, dependent consolidation, and/or severe hypoxemia	Triggering insult such as sepsis, trauma, aspiration, transfusion, and/or severe infection
Cardiogenic pulmonary edema	Perihilar edema, septal thickening, pleural effusions, and/or vascular congestion with often cardiomegaly	Elevated BNP, cardiac history, reduced ejection fraction, and/or response to diuresis
Diffuse alveolar hemorrhage	Ground-glass opacities and/or consolidation, which are often diffuse or central	Anemia, autoimmune disease, vasculitis, anticoagulation, and/or bronchoscopy findings (Note that hemoptysis may be absent)
Leptospirosis	Multifocal ground-glass opacities, consolidation, pulmonary hemorrhage, and/or possible pleural effusions	Freshwater/animal urine exposure, jaundice, renal failure, thrombocytopenia, and/or conjunctival suffusion
HFRS/nephropathia epidemica	Renal enlargement, perirenal fluid, and/or retroperitoneal edema	Prominent AKI, proteinuria, hematuria, abdominal/flank pain, and/or endemic exposure

### Infection control and clinical management in the radiology department

#### Patient precautions and respiratory protection

The management of patients with suspected hantavirus infection requires a tiered approach to infection control that is tailored to the specific viral strain and clinical presentation. The World Health Organization (WHO) and the Centers for Disease Control and Prevention (CDC) recommend Standard Precautions for most hantaviruses, as the primary route of infection is through the inhalation of aerosolized rodent excreta rather than horizontal human transmission [3, 18]. However, given the documented person-to-person transmission of ANDV, both the WHO and CDC advise escalating to droplet and contact precautions when ANDV is suspected, particularly in the context of recent travel to South America or the 2026 maritime clusters [1, 4, 15]. Broader radiology workflow measures, such as minimizing transport, using dedicated routes, and applying room downtime when indicated, should be understood as institutional respiratory-pathogen practices adapted to the acute setting rather than hantavirus-specific requirements in every case. This necessitates the use of fluid-resistant gowns, gloves, and medical masks in the radiology suite. For aerosol-generating procedures (AGPs) or in high-risk environments, the use of NIOSH-approved N95 respirators (or FDA-cleared surgical N95s) or higher-level respirators are essential [7, 18, 19].

### Clinical handling and triage

Effective management in the Emergency Radiology department centers on rapid identification and the minimization of patient movement. Suspected patients should be masked immediately upon arrival and transitioned to a private room or a negative-pressure airborne infection isolation room (AIIR) may be appropriate when aerosol-generating procedures are anticipated, when ANDV is suspected with significant respiratory symptoms, or when required by institutional protocols for undifferentiated severe respiratory infection [7]. Portable imaging is strongly preferred over transporting the patient to the radiology department to prevent cross-contamination of high-traffic areas [7, 8]. If the patient must be brought to the imaging suite, they should follow a dedicated path, and the department should coordinate to ensure the room is cleared of other patients and non-essential staff [7, 19].

### Environmental cleaning and equipment decontamination

Hantaviruses are enveloped viruses and are highly susceptible to most EPA-registered hospital-grade disinfectants, including 10% bleach solutions, alcohol-based cleaners, and phenolic compounds [3, 18]. Following the imaging of a suspected case, all “high-touch” surfaces, including the gantry, imaging tables, and lead shielding, must be thoroughly disinfected. The CDC suggests that the duration of room “down-time” or isolation post-procedure should follow the same air-exchange calculations used for other respiratory pathogens (e.g., SARS-CoV-2), typically allowing for 99.9% air filtration based on the facility’s air changes per hour (ACH) [7, 20]. In many modern radiology suites, this isolation period ranges from 30 to 60 minutes before the room can be safely reused for another patient [7, 20].

### Ethical considerations

Hantavirus outbreaks impact more than just clinical care. These outbreaks raise important ethical questions and challenge healthcare systems. The COVID-19 pandemic highlighted how quickly critical care resources like ECMO machines, ICU beds, and trained staff can become scarce during surges of severe respiratory illness [1, 21]. Furthermore, it highlighted how important it is to keep health-care workers safe. While hantaviruses usually don’t spread as easily between people as SARS-CoV-2, the Andes virus makes robust infection-control practices and clear isolation guidelines essential [4, 15]. These considerations also support a “One Health” framework, in which environmental exposure and public health surveillance are integrated to improve outbreak detection.

## Conclusion

For emergency medicine and radiology teams, HPS moves quickly and brings tough challenges. Early recognition of characteristic imaging findings can help doctors get patients to the ICU and start advanced care before their lungs fail [3, 7]. Late diagnosis can overwhelm emergency and critical care services especially in places with limited resources. These issues also highlight the value of a “One Health” approach, which connects human, animal, and environmental surveillance to strengthen preparedness and access to diagnostics across different regions [1, 5].

## Data Availability

Data sharing is not applicable to this article, as no datasets were generated or analyzed during the current study.
